# Characterization of Endothelial Progenitor Cell Interactions with Human Tropoelastin

**DOI:** 10.1371/journal.pone.0131101

**Published:** 2015-06-26

**Authors:** Young Yu, Steven G. Wise, Praveesuda L. Michael, Daniel V. Bax, Gloria S. C. Yuen, Matti A. Hiob, Giselle C. Yeo, Elysse C. Filipe, Louise L. Dunn, Kim H. Chan, Hamid Hajian, David S. Celermajer, Anthony S. Weiss, Martin K. C. Ng

**Affiliations:** 1 Department of Cardiology, Royal Prince Alfred Hospital, Sydney, NSW, 2050, Australia; 2 The Heart Research Institute, Sydney, NSW, 2042, Australia; 3 Sydney Medical School, University of Sydney, Sydney, NSW, 2006, Australia; 4 School of Molecular Bioscience, University of Sydney, Sydney, NSW, 2006, Australia; 5 Bosch Institute, University of Sydney, Sydney, NSW, 2006, Australia; 6 Charles Perkins Centre, University of Sydney, Sydney, NSW, 2006, Australia; University of Torino, ITALY

## Abstract

The deployment of endovascular implants such as stents in the treatment of cardiovascular disease damages the vascular endothelium, increasing the risk of thrombosis and promoting neointimal hyperplasia. The rapid restoration of a functional endothelium is known to reduce these complications. Circulating endothelial progenitor cells (EPCs) are increasingly recognized as important contributors to device re-endothelialization. Extracellular matrix proteins prominent in the vessel wall may enhance EPC-directed re-endothelialization. We examined attachment, spreading and proliferation on recombinant human tropoelastin (rhTE) and investigated the mechanism and site of interaction. EPCs attached and spread on rhTE in a dose dependent manner, reaching a maximal level of 56±3% and 54±3%, respectively. EPC proliferation on rhTE was comparable to vitronectin, fibronectin and collagen. EDTA, but not heparan sulfate or lactose, reduced EPC attachment by 81±3%, while full attachment was recovered after add-back of manganese, inferring a classical integrin-mediated interaction. Integrin α_V_β_3_ blocking antibodies decreased EPC adhesion and spreading on rhTE by 39±3% and 56±10% respectively, demonstrating a large contribution from this specific integrin. Attachment of EPCs on N-terminal rhTE constructs N25 and N18 accounted for most of this interaction, accompanied by comparable spreading. In contrast, attachment and spreading on N10 was negligible. α_V_β_3_ blocking antibodies reduced EPC spreading on both N25 and N18 by 45±4% and 42±14%, respectively. In conclusion, rhTE supports EPC binding via an integrin mechanism involving α_V_β_3_. N25 and N18, but not N10 constructs of rhTE contribute to EPC binding. The regulation of EPC activity by rhTE may have implications for modulation of the vascular biocompatibility of endovascular implants.

## Introduction

Circulating endothelial progenitor cells (EPCs) [1] are increasingly recognized to play an important role in cardiovascular regeneration. Increased levels of EPCs correlate with reduced risk of cardiovascular mortality [2] and contribute to angiogenesis, vasculogenesis [3] and the repair of injured vasculature [4]. EPCs are mobilized from the bone marrow and other sites in response to cytokines, growth factor stimulation, and ischemia [5]. EPCs have also shown therapeutic potential in vascular medicine. When EPCs were expanded *ex vivo* and injected into pre-clinical models of hind limb ischemia [6] and myocardial infarction [7], a reduction in ischemia, improved limb salvage and myocardial function has been observed.

Accordingly, there is increasing interest in the potential for EPCs to facilitate re-endothelialization of endovascular prostheses such as stents following implantation in the vasculature [5]. The deployment of metallic stents results in significant injury to the vessel wall and disruption of the endothelium, predisposing them to thrombosis and neointimal hyperplasia from excessive smooth muscle proliferation [8]. Rapid re-endothelialization following vascular injury attenuates neointimal hyperplasia [9] while also deterring thrombus formation [10]. It is increasingly recognized that circulating EPCs substantially contribute to vascular prosthesis endothelialization [11,12], making them important mediators of implant compatibility. However, in animal models of induced vascular injury the use of pharmacological stimulation [13,14] or infusion of endothelial-like mononuclear cells has been met with variable success [15,16], perhaps reflecting the limitations of a systemic, less locally targeted approach.

Local enhancement and modulation of EPC activity at the site of injury is a potential strategy for improving the clinical efficacy of vascular implants. A range of sub-endothelial vascular matrix biomolecules have been shown to play critical roles in local regulation of thrombosis, endothelialization and smooth muscle cell proliferation, making these attractive candidates for modulation of vascular biocompatibility [17]. Elastin, an abundant elastic tissue protein, imparts both elasticity and important cell signaling. In elastin knockout mice, fatal arterial occlusive disease results from uncontrolled proliferation of vascular smooth muscle cells (VSMCs), suggesting a critical role for elastin in VSMC inhibition [18], consistent with recent *in vitro* findings [19]. Elastin also promotes endothelial cell activity [20] while having low thrombogenicity [21]. These properties were mirrored when rhTE, the soluble pre-cursor of elastin was immobilized on metallic substrates, enhancing endothelial growth [22] and reducing thrombogenicity [23].

Elastin has several established modes of cell interaction, including via: 1) the elastin binding protein, which binds to GXXPG consensus sequences [24]; 2) cell surface glycosaminoglycans at the C-terminus [25] and; 3) the integrin α_V_β_3_, through the very last amino acids of the monomer, GRKRK [26]. These binding assays have predominantly been carried out using the elastin monomer, tropoelastin and fibroblasts. Recently, an additional integrin-mediated fibroblast binding site for tropoelastin has been described, encompassing domains 17 and 18, in the central region of the protein [27]. Tropoelastin interaction with endothelial cells appears to be somewhat different, supporting attachment and proliferation with as little as the first 10 domains (N10) [28].

In this study we sought to characterize endothelial progenitor cell interactions with recombinant human tropoelastin (rhTE). We used outgrowth endothelial progenitor cells (OECs), arising from cultured peripheral mononuclear cells [29], increasingly recognized to most closely conform to the definition of a ‘true EPC’ [29]. OECs are characterized by robust proliferative capacity and high sensitivity to angiogenic stimuli [30], incorporate into repairing endothelium, and can directly form vascular tubules [29,31,32]. In studies involving bone marrow transplant patients, cultured OECs had the genotype of the donor rather than the recipient, consistent with a bone marrow origin for these cells [33]. OECs are herein referred to as EPCs.

We report that rhTE can support EPC attachment, spreading, and proliferation. Attachment and spreading occur via an integrin-mediated mechanism, involving but not limited to the α_V_β_3_ integrin. Using truncation constructs of N-terminal domains we show that N25 and N18 can support EPC attachment and proliferation similar to full-length rhTE, while binding to N10 is poor.

## Materials and Methods

### Materials

**Fig 1:**
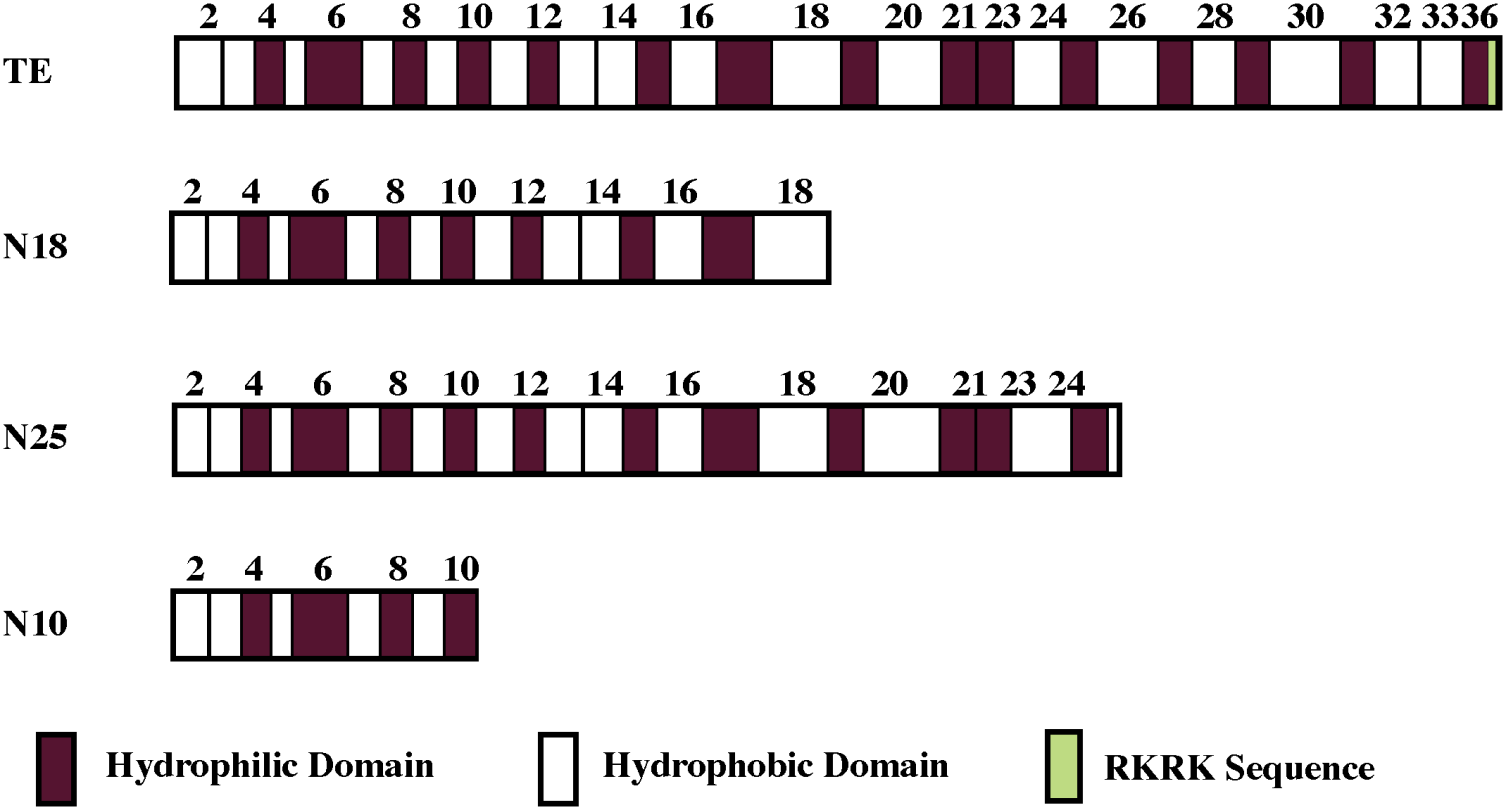
Schematic diagram showing full-length rhTE and constructs N18, N25 and N10. Key domain features are indicated.

Full-length recombinant human tropoelastin (rhTE) and N-terminal constructs representing the first 25 (N25), 18 (N18), and 10 (N10) domains (Fig 1) of rhTE were produced in a previously described *E*. *coli* expression system [34]. Human dermal fibroblasts (HDF; line GM3348) were obtained from Coriell Research Institute (Camden, NJ, USA). Primary human coronary artery smooth muscle cells (SMCs) were obtained from Cell Applications (San Diego, CA, USA). Fibronectin and vitronectin from human plasma, as well as calf skin collagen type 1 (Sigma) were used as controls. Anti-human integrin antibodies, α_v_β_3_-clone LM609, α_5_β_1_-clone JBS5, and α_2_β_1_-clone BHA2.1 were obtained from Millipore. All other reagents were purchased from Sigma.

### EPC isolation and culture

This study had ethics committee approval in accordance with the Declaration of Helsinki and Good Clinical Practice Guidelines (Sydney South West Area Health Service Ethics Approval #X06-0191). All study participants provided written informed consent. EPCs were isolated from 100–120 ml adult peripheral blood taken from healthy young male volunteers as previously described [31], collected into EDTA tubes [33]. Mononuclear cells (MNCs) were isolated using Lymphoprep, a density gradient separation. MNCs contained in the buffy coat were aspirated and resuspended in EGM-2 media. 1–2 x 10^7^ cells/ml were seeded onto 6-well type I rat tail collagen pre-coated plates. After 24 h, each well was gently washed to remove non-adherent cells. Media were exchanged daily for the first 7 days and then subsequently every second day. EPCs emerge as cobble stone shaped clusters of cells between days 14–21 [33]. EPC colonies reaching 80% confluence of the wells were carefully extracted and expanded in T25 flasks and subsequently moved to T175 flasks. All EPCs used were at passages 5–10.

### Immunophenotyping

Confirmation of EPC phenotype was assessed using established flow cytometry methods, complemented with representative imaging [31]. For characterization using flow cytometry, OECs were detached, washed in PBS and resuspended in 0.5% BSA before blocking with FcR Blocking Reagent (Miltenyi Biotec) and staining with CD34-PerCP-Cy5.5, CD45-APC-Cy7, CD31-PE, CD14-APC, CD309(VEGFR2)-PE, CD54-PE for 30 min at 4°C (BD Pharmingen). The cells were then washed with PBS and stained with Zombie Aqua Viability dye (BioLegend) for 30 min at room temperature in PBS, before undergoing a final wash and resuspension in 0.5% BSA for reading on the FACSVerse (BD Biosciences, San Jose, California, USA). Unstained cells and fluorescence minus one (FMO) analyses was used for negative controls. Endothelial cell specific uptake of acetylated low density lipoprotein (Dil-Ac-LDL) and isothiocyanate-Ulex europaeus agglutinin I lectin (FITC-Ulex) binding was assessed. The presence of CD31 was also determined using a CD31/PECAM1 antibody (1:100 in antibody diluent; DAKO S3022). Fixed cells had their membranes permeablized with 1% Triton-x-100 in PBS for 5 min, before blocking with peroxidase (30 min) and normal goat serum (1 h). HRP secondary antibody anti-mouse kit (DAKO K4000) was used for detection. Cell nuclei were counterstained with haematoxylin. 10 random fields of view per slide at 20X magnification were captured on a Zeiss Axiovision Imager Z2 and representative images shown. Exposures were equal for comparative images.

### N-terminal specific ELISA

The relative amount of tropoelastin constructs bound to cell culture wells was determined with an ELISA. Triplicate wells were coated with 40 μg/mL of N10, N18, N25 or rhTE at 4°C overnight. Unbound tropoelastin was removed with three PBS washes. Non-specific antibody binding was blocked with 3% (w/v) BSA for 1h at room temperature, and excess BSA was washed off with PBS. Surface-bound tropoelastin was detected with a 1:5000 dilution of a custom-made mouse antibody specific against tropoelastin domain 6 (Abmart) for 1h at room temperature. Wells were washed three times with PBS and incubated with a 1:5000 dilution of goat anti-mouse IgG conjugated with horseradish peroxidase. Unbound secondary antibody was removed with four PBS washes, while the bound species was visualized by incubation with ABTS solution (1.04 mg/mL ABTS, 0.05% (v/v) H_2_O_2_, 10 mM CH_3_COONa, 5 mM Na_2_HPO_4_) at 37°C for 1h. Sample absorbance were read at 405 nm with a plate reader, and subtracted by the absorbance of ABTS-only controls.

### Cell adhesion

Fibronectin (FN) and collagen (CN) (5 μg/ml) were used as positive controls with concentrations based on dose-attachment/spreading titration assays. For both FN and CN, attachment and spreading of EPCs peaked at a concentration of 1.5 μg/ml; no significant increases were seen at concentrations up to 40 μg/ml (data not shown). A dose of 5 μg/ml for FN and CN was used to ensure plateau biological response. 100 μl of rhTE and truncated constructs were diluted in phosphate buffer saline (PBS), added to 96-well tissue culture plates and left overnight at 4°C. Unbound protein was aspirated and washed with PBS followed by addition for 1 h at room temperature of 150 μl 1% (w/v) heat-denatured bovine serum albumin (BSA). EPCs were trypsinized and adjusted to a cell density of 2–3 x 10^5^ cells/ml in serum-free EGM-2 medium. Unbound BSA was removed from wells by PBS washing, then 100 μl of cells were added to each well, and allowed to attach for 60 min at 37°C. Non-adherent cells were washed off and adherent cells were fixed with 3.3% formaldehyde for 30 min. Attached cells were quantified following crystal violet staining; the stain was released with addition of 100 μl of 10% (w/v) acetic acid, and the absorbance was read at 580 nm. A standard curve was generated with defined cell numbers.

### Cell spreading

Tissue culture plates were prepared in a similar manner to cell attachment. EPCs were harvested and made to a density of 1x10^5^ cells/ml in serum-free EGM-2. 100 μl of cells were added to the wells and allowed to spread over 2 h at 37°C, after which 10 μl per well of 37% formaldehyde was directly added to the cells for 30 min. Using phase contrast microscopy, cells were considered unspread if they were small, round, and phase-bright. Spread cells were larger, phase dark with irregular cytoplasmic projections.

### Cell proliferation

500 μl of protein (rhTE 40 μg/ml, vitronectin 0.5 μg/ml, CN 5 μg/ml and FN 5 μg/ml) and N-terminal domain constructs (40 μg/ml) in PBS were sterile-filtered (0.22 μm filters) and coated overnight at 4°C onto wells of 24-well plates. Wells were washed 3 times with 500 μl PBS, followed by blocking for 1 h with 600 μl of 1% BSA. Excess BSA was removed by washing with PBS. EPCs were prepared as described above except that cells were suspended in EBM-2 with 10% fetal calf serum to a density of 6x10^3^ cells/ml. 1 ml of cell suspension was added to each well. Cells were left to proliferate for either 3 or 5 days, after which they were fixed with 3.3% formaldehyde and quantified using crystal violet [26].

### Inhibition of EPC adhesion

The effect of lactose, heparan sulfate, and EDTA on EPC attachment was determined as described except that after BSA blocking 50 μl each at double-concentration were added to wells followed by an equal volume of 4x10^5^ cells/ml of EPCs suspended in serum free EGM-2 medium.

### Divalent cation add-back

After the wells were blocked with BSA, 50 μl of calcium (Ca^2+^), magnesium (Mg^2+^) or manganese (Mn^2+^) was added at double-concentration and mixed with equal volume of 4x10^5^ cells/ml EPCs resuspended in cation-free HEPES buffer.

### Anti-integrin antibody assays

EPCs were suspended at 2x10^5^ cells/ml density and aliquoted in to microcentrifuge tubes. Integrin-blocking antibodies were added to the tubes to a concentration of 30 μg/ml and left at 37°C for 1 h. 100 μl of antibody and cell suspension was added to BSA-blocked wells coated with rhTE or N-terminal constructs. Adhesion, spreading and proliferation assays were as described.

### Statistical Methods

Data are presented as mean ± SEM. Means were compared using 1-way analysis of variance (ANOVA) with pairwise comparisons (Tukey’s multiple comparisons test). Statistical significance was defined as p<0.05.

## Results

### Characterization of EPC phenotype

**Fig 2:**
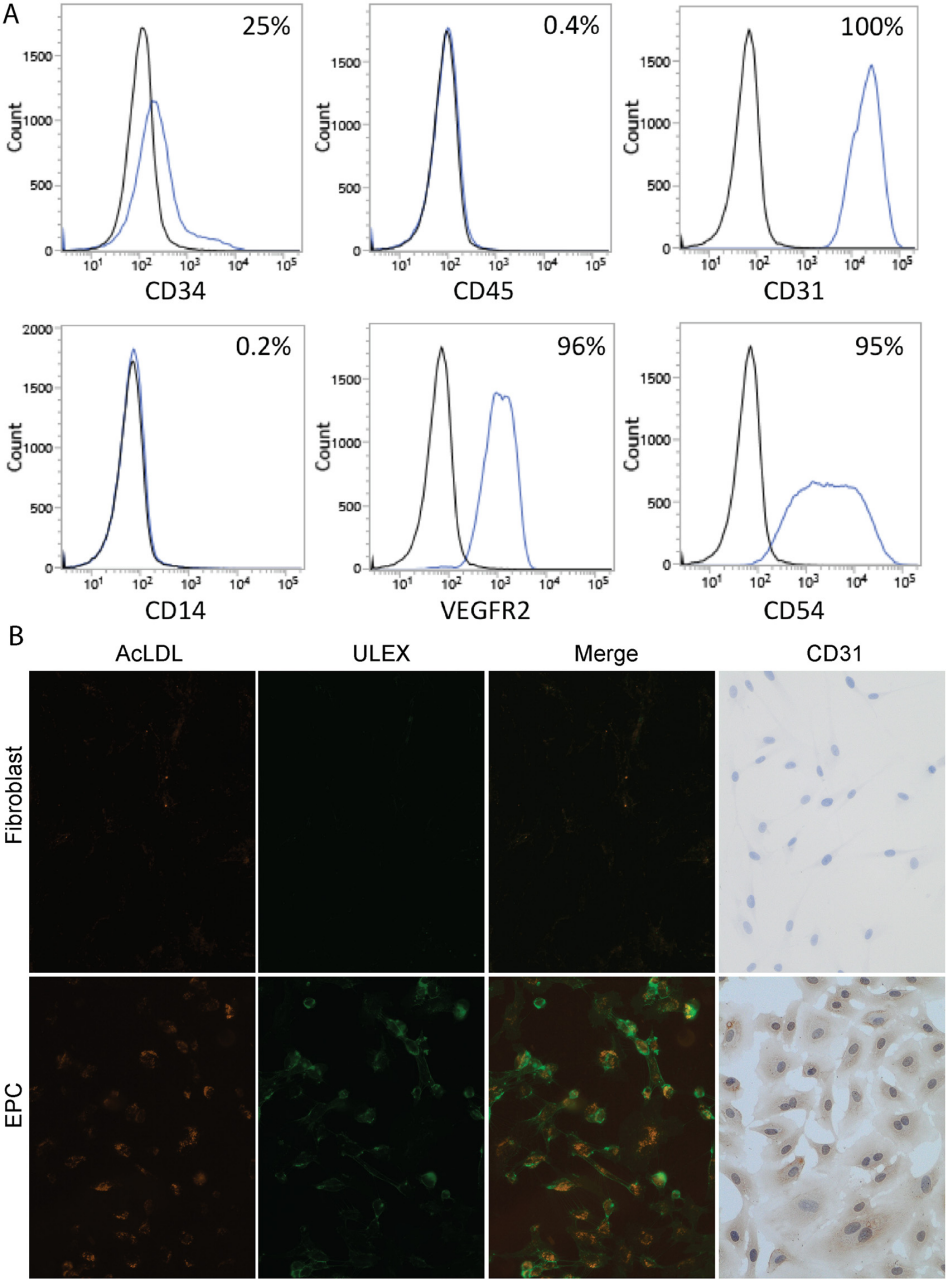
OEC characterization by flow cytometry. A) Stained cells are shown as blue histograms, while unstained controls are shown in black. The percentage of positive cells is shown in the top right of each graph. The OECs are CD34/31/54/VEGFR2 positive and CD45/14 negative. B) Representative images of the binding of isothiocyanate-Ulex europaeus agglutinin I lectin binding (ULEX), uptake of acetylated low density lipoprotein (AcLDL) and staining for CD31 by EPCs (bottom row of panel) but not by fibroblasts (top row of panel). Together, these results are indicative of a positive endothelial cell phenotype.

Consistent with previous studies [31], colonies of EPCs with a cobblestone morphology emerged from peripheral MNCs between days 14–21. Characterization by flow cytometry suggests that the OECs used in these experiments are of endothelial lineage (CD31 99.7±0.1%; CD54 95.4±0.1%; VEGFR2 96.3±0.1% positive) with no hematopoietic cells present (CD45 0.43±0.03%; CD14 0.20±0.03% negative). A subset of cells also still express CD34 (25.2±0.5%), a progenitor cell marker. In contrast to human dermal fibroblasts, EPCs displayed characteristic Ulex binding and incorporation of acLDL, and stained positively for CD31 (Fig 2).

### Tropoelastin enhances EPC attachment, spreading and proliferation

**Fig 3:**
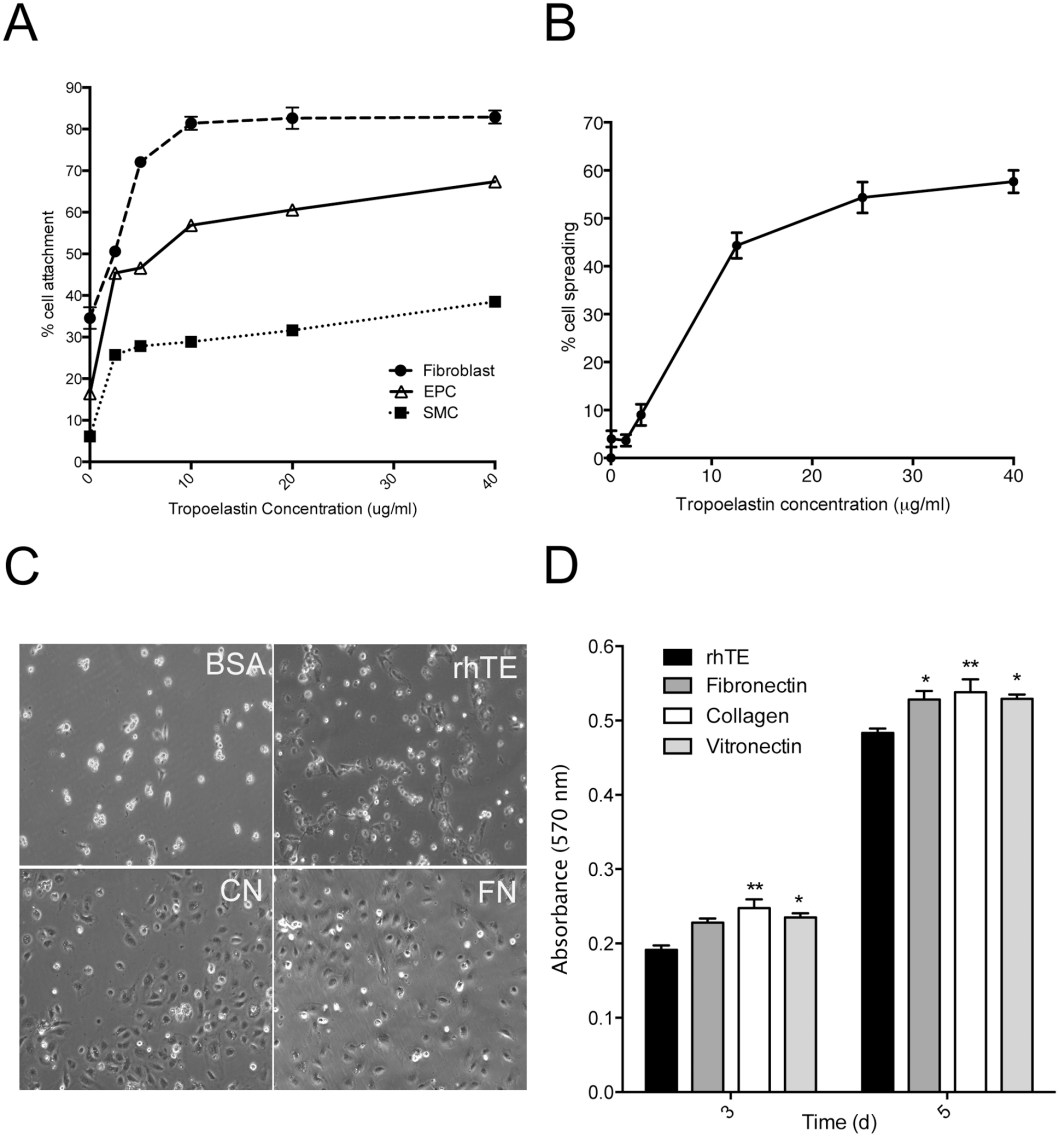
Cell binding to recombinant human tropoelastin. (A) Relative attachment of human dermal fibrolasts, EPCs and human coronary artery smooth muscle cells (SMC) to increasing concentrations of tropoelastin. (B) The percentage of spread EPCs on increasing concentrations of tropoelastin. (C) Phase contrast microscopy of spreading EPCs on BSA-blocked wells, tropoelastin (rhTE) collagen (CN) fibronectin (FN). Images were taken at 10x magnification. (D) EPC proliferation on days 3 and 5, respectively. Error bars represent S.E.M. of triplicate measurements.

EPC attachment to rhTE was concentration dependent, reaching 57±1% at 10 μg/ml, but a maximum of 67±1% at 40 μg/ml, the highest concentration tested (Fig 3A). For context, attachment of human dermal fibroblasts and human coronary artery smooth muscle cells (SMC) to rhTE was also examined in the same assay. Fibroblast attachment was greatest, reaching 83±2% at the highest tropoelastin concentration, in contrast to SMCs which only had 39±1% of cells attached. rhTE also enhanced EPC spreading in a concentration-dependent manner reaching 54±3% at 25 μg/ml and 58±2% at 40 μg/ml. Spreading was not significantly different between 25 and 40 μg/ml coating concentrations (p = 0.92, Fig 3B).

EPC attachment was higher on FN (5 μg/ml) and CN (5 μg/ml) at 77±3% and 73±1%, respectively (p<0.01 vs. rhTE, data not shown). EPC spreading was also greater on FN (77±3%) and CN (82±6%, p<0.01 vs. rhTE), demonstrated in representative phase contrast images (Fig 3C).

For cell proliferation studies, EPCs were seeded on rhTE and compared to vitronectin, collagen (CN) and fibronectin (FN) coated surfaces. Cells were quantified with crystal violet staining and expressed as relative absorbance at 570 nm. On day 3, EPC proliferation on rhTE was not significantly different to FN (Fig 3D). The greater proliferation of EPCs on vitronectin (p<0.05) and collagen (p<0.01) did reach significance compared to rhTE, but were indistinguishable from each other. By day 5, EPCs grown on rhTE had increased 152±4%, relative to day 3. EPCs on vitronectin, collagen and FN were all significantly more abundant than on rhTE.

### EPC binding to tropoelastin involves integrin α_V_β_3_

**Fig 4:**
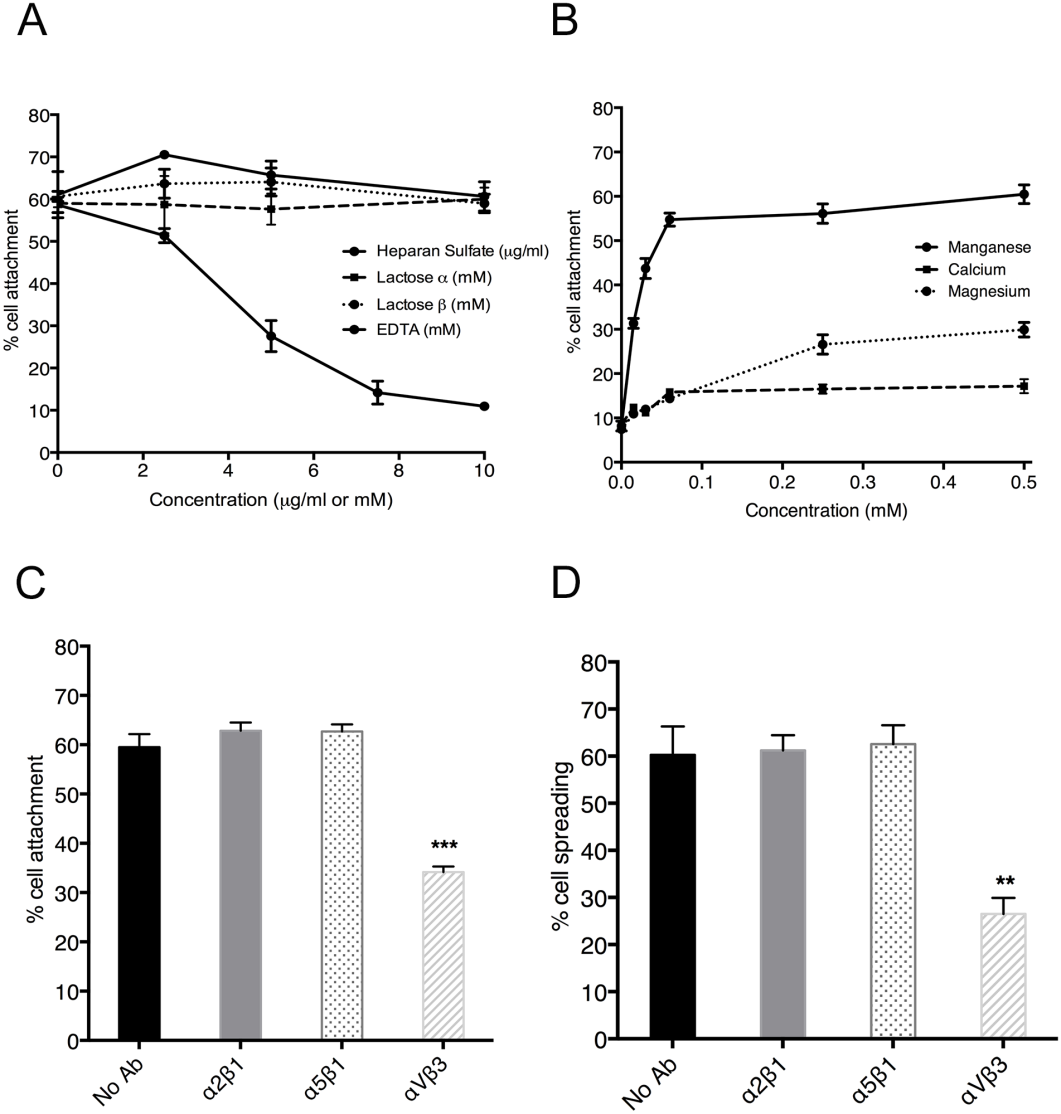
Mechanism of EPC attachment to rhTE. (A) EPCs attached to 40 μg/ml rhTE in the presence of α-lactose, β-lactose, heparan sulfate, or EDTA. (B) Attachment of EPCs to 40 μg/ml rhTE in the presence of Ca^2+^, Mg^2+^, or Mn^2+^. (C) and (D) Inhibition of EPC attachment and spreading on 40 μg/ml rhTE using antibodies to integrins α_2_β_1_, α_5_β_1_, and α_v_β_3_. Error bars represent S.E.M. of triplicate measurements.

Cells interact with tropoelastin through three main mechanisms, i) cell surface glycosaminoglycans (GAGs) ii) transmembrane elastin binding protein, and iii) integrins. These interactions are inhibited *in vitro* by administration of heparan sulfate, lactose and EDTA respectively [26]. We explored the nature of the interaction between EPCs and rhTE using this approach. Heparan sulfate up to 10 μg/ml caused no reduction in EPC attachment to rhTE. The inclusion of α- and β-lactose up to 10 mM also had no effect on EPC adhesion. In contrast, we saw a progressive reduction of EPC adhesion with increasing concentrations of EDTA. At 10 mM EDTA there was an 81±3% (p<0.001) reduction in attachment (Fig 4A), suggesting an integrin-mediated interaction.

Divalent cation add-back experiments provided further evidence of integrin binding [35]. In the absence of cations, EPC adhesion was similar to background levels, while a concentration-dependent increase in EPC attachment was evident in the presence of manganese. Full recovery was seen with 0.25 mM manganese. In contrast, there was only a small increase in EPC attachment with calcium up to 0.5 mM. Magnesium had an intermediate effect (Fig 4B).

Anti-α_5_β_1_ and anti-α_2_β_1_ integrin blocking antibodies were found to have no effect on EPC adhesion, while anti-α_V_β_3_ antibodies led to a 39±3% (p <0.001) reduction in EPC attachment (Fig 4C). A similar effect on EPC spreading was evident with the inclusion of anti-α_V_β_3_ antibody leading to a 56±10% (p<0.01) decrease in cell spreading. Anti-α_2_β_1_ and -α_5_β_1_ antibodies had no impact on EPC spreading (Fig 4D). No effect on proliferation was observed in the presence of integrin blocking antibodies (S1 Fig).

### EPC attachment, spreading, and proliferation are supported by N-terminal domains of tropoelastin

**Fig 5:**
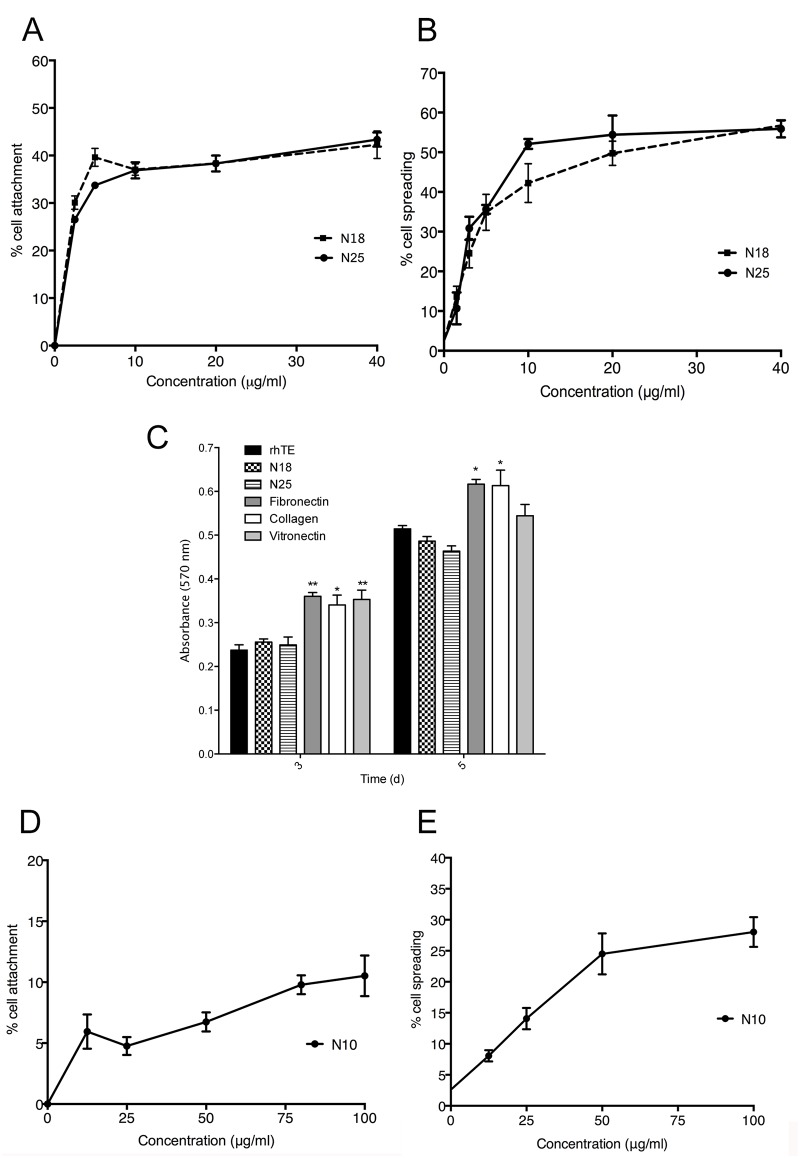
EPC attachment (A) and spreading (B) on tropoelastin constructs N25 and N18. C) and (D) Proliferation of EPCs on N25 and N18 constructs, on days 3 and 5, respectively. Values were normalized to proliferation on collagen. Error bars represent S.E.M. of triplicate measurements. EPC attachment (E) and spreading (F) to tropoelastin construct N10. Attachment and spreading assays on N10 constructs was done on the same cell plates as for N25 and N18 constructs. Results are expressed separately to highlight higher maximal concentrations of N10 used. Error bars represent S.E.M. of triplicate measurements.

The C-terminus of rhTE is known to be important for adhesion of fibroblasts [26,36]. An additional contribution from the mid-domain regions 17/18 has only been recently described [27]. In contrast, endothelial cell binding to rhTE can be supported by the first 10 N-terminal domains [28]. In this study we sought to determine a preferred binding region for EPCs to rhTE. We expressed and purified truncation constructs of rhTE, spanning regions from the N-terminus to domains 25 (N25), 18 (N18), and 10 (N10). An ELISA specific for domain 6 of tropoelastin showed no significant differences in the relative amount of each present on tissue culture plastic (S2 Fig). N25 supported 38±2% attachment of EPCs at a concentration of 20 μg/ml and up to 43±3% at 40 μg/ml. A comparable level of 38±2% attachment was seen on N18 at 20 μg/ml, and up to 42±5% at 40 μg/ml (N25 vs. N18, p = 0.09) (Fig 5A). EPC spreading was comparable on N25 and N18 at 56±2% and 57±1%, respectively (N25 vs. N18, 40μg/ml, p = 0.92) (Fig 5B). When compared to full length rhTE EPC spreading on N25 and N18 constructs was similar (rhTE vs N25 and N18, P = 0.81 and P = 0.94, respectively).

Proliferation of EPCs on N18 and N25 on day 3 was not significantly different to each other or rhTE, but less than FN (p<0.01), CN (p<0.05) and vitronectin (p<0.01). (Fig 5C). On day 5, proliferation increased on rhTE (117±4%), N18 (91±3%) and N25 (86±3%,) relative to day 3 and again were not significantly different to each other. FN and CN remained statically superior, but vitronectin was equivalent at this time. In contrast, N10 displayed poor EPC interaction, even with an increased concentration up to 100 μg/ml. A maximum cell attachment of 11±2% (Fig 5E) and spreading of 28±2% (Fig 5F) was observed.

**Fig 6:**
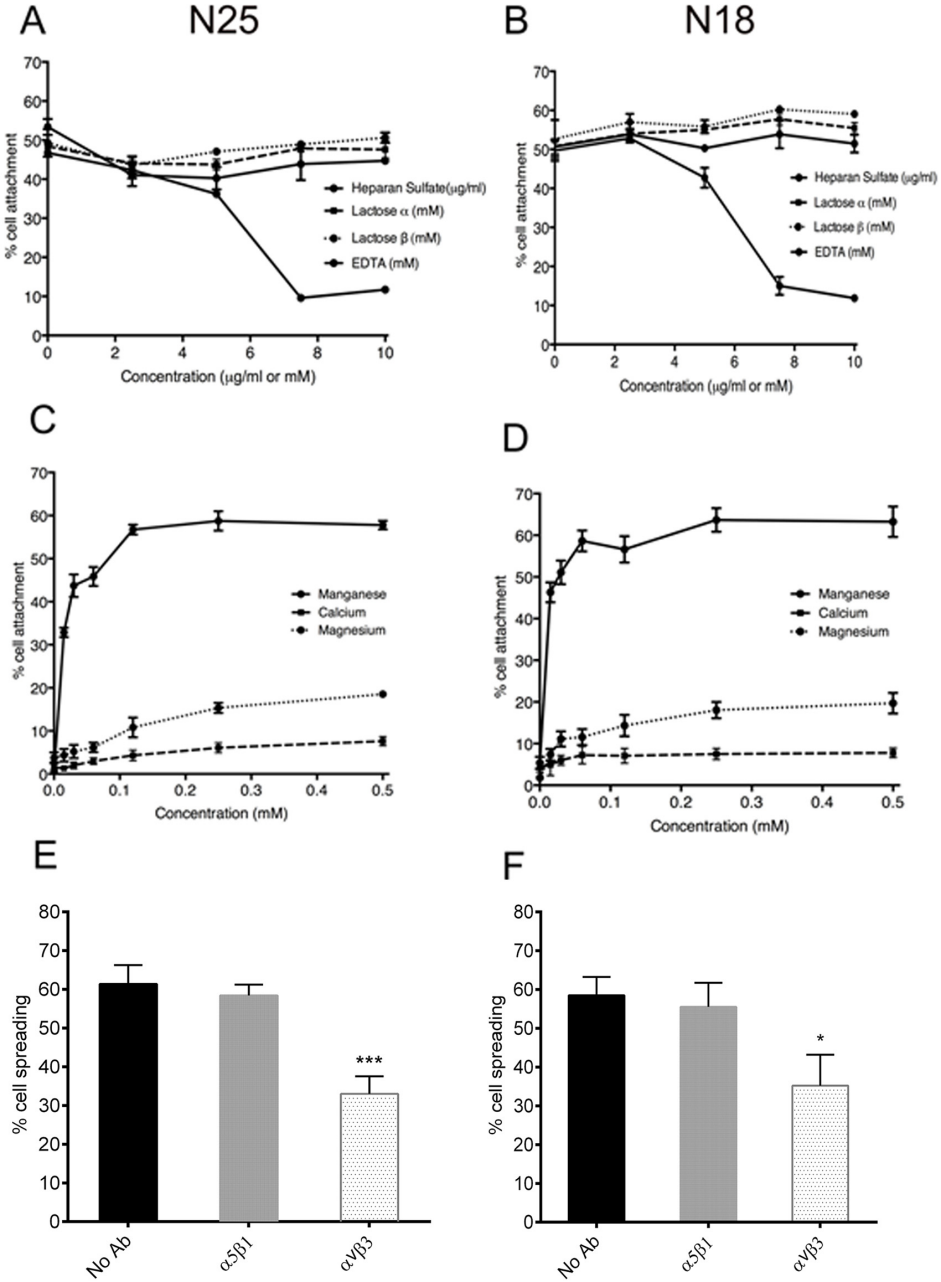
Mechanism of EPC attachment to truncated tropoelastin constructs. (A) and (B) EPCs attached to 40 μg/ml N25 and N18 respectively, in the presence of α-lactose, β-lactose, heparan sulfate or EDTA. (C) and (D) Attachment of EPCs to 40 μg/ml N25 and N18, respectively, in the presence of Ca^2+^, Mg^2+^, or Mn^2+^. (E) and (F), Inhibition of EPC spreading on 40 μg/ml N25 and N18 respectively, using antibody that inhibits binding to integrin α_v_β_3_. Error bars represent S.E.M. of triplicate measurements.

As for rhTE, we investigated the mechanism of interaction between N25 and N18 constructs with EPCs. No appreciable effect on cell attachment was seen in the presence of α-lactose, β-lactose, and heparan sulfate. However, the addition of EDTA led to a 65±2% (p<0.0001), and 76±5% (p<0.001) reduction in attachment to N25 and N18, respectively (Fig 6A and 6B). When divalent cations were re-introduced to cation-free buffer, a dose-dependent full recovery of EPC attachment to both N25 and N18 was seen with manganese (Fig 6C and 6D), but not for magnesium or calcium, as expected for an integrin interaction. Anti-α_V_β_3_ antibody led to a 45±4% (p<0.001) and 42±14% (p<0.05) decrease in EPC spreading on N25 and N18, respectively (Fig 6E and 6F), while anti-α_5_β_1_ had no effect. These results collectively establish an integrin-based mode of EPC interaction with N25 and N18 that involves α_V_β_3_.

## Discussion

In this study we sought to characterize the interaction of EPCs with recombinant human tropoelastin. We found that EPCs in the form of outgrowth endothelial cells, attached to and spread on rhTE, similar to previous findings for human umbilical vein endothelial cells [19]. The level of attachment of EPCs was intermediate, between fibroblasts, well characterized to adhere strongly to rhTE, and SMCs, which were expected to have low adhesive capacity. FN and CN demonstrated higher levels of cell adhesion, consistent with previous studies [37,38]. However, the role of FN and CN for clinical applications is hindered by their inherent thrombogenicity, platelet activation [39,40], and chemoattraction for VSMCs [19]. Proliferation of EPCs on rhTE was statistically less than vitronectin, CN and FN at days 3 and 5 but was substantial overall. These findings establish that rhTE supports EPC attachment, spreading and proliferation.

The elastin binding protein [41,42] mediates the chemotactic properties of elastin but cell attachment is increasingly thought to be through alternate mechanisms. The C-terminus is known to be a strongly cell adhesive region in tropoelastin. Bovine chondrocytes adhere to the C-terminal 29–36 domains of bovine elastin through surface glycosaminoglycans (GAGs) [25] whereas human dermal fibroblasts interact with rhTE through the C-terminal motif (GRKRK) using an integrin-based mechanism [26]. A second fibroblast binding site comprising domains 17 and 18 also appears to be integrin-mediated [27]. Human umbilical vein endothelial cells however bind preferentially to the N-terminus [28], suggesting the possibility of differential binding to tropoelastin dependent on cell type.

We first identified that the interaction between human EPCs and rhTE is likely integrin mediated. Inhibitor studies indicated that while EPC adhesion to rhTE was not through the elastin binding protein or cell surface GAGs, multiple experiments support an integrin-based mechanism. These included both a dose-dependent decrease in EPC attachment to rhTE with increasing concentrations of EDTA and a dependence on divalent cations and specifically manganese. Manganese induces strong ligand affinity in integrin α_V_β_3_ [43]. Using an inhibitory antibody to integrin α_V_β_3_ we saw a decrease in both EPC adhesion and spreading on rhTE. The extent of inhibition was less than that previously seen with fibroblasts [26], but consistent with levels seen for mature endothelial cells, indicating that additional integrins may be involved [44]. Antibodies to α_2_β_1_ and α_5_β_1_ had no effect on either EPC attachment or spreading. α_2_β_1_ typically interacts with CN via GFOGER [45] and α_5_β_1_ binds FN through an RGD motif [46]; rhTE contains neither motif [47].

Conflicting information exists for the capacity of the N-terminus to support cell attachment [19,25]. Recently N-terminal constructs of rhTE were shown to support the adhesion and growth of mature endothelial cells [28] indicating that, in addition to the C-terminus, other cell binding site(s) are present in rhTE. In this study we used progressively shorter truncation domains of the N-terminus to investigate support for EPC adhesion in each region.

The N25 construct accounted for the largest proportion of cell attachment (68±5%, p<0.001 of full length rhTE) and showed spreading (93±3%) that was comparable to that seen on full-length rhTE. Further truncation to the shorter N18 construct however presented comparable attachment, spreading and proliferation to N25, suggesting a model where the region between domains 18 and 25 is not required for cell adhesion. The even shorter N10 construct consistently showed reduced cell attachment and spreading that was only marginally higher than background BSA levels, even at high concentrations. Together these results suggest that EPCs bind a region bracketed by domains 10 and 18 of rhTE, consistent with the additional binding site recently described for fibroblasts [27].

In conclusion, rhTE supports EPC binding via an integrin mechanism involving α_V_β_3_ and interacting with tropoelastin between domains 10 and 18. These findings suggest that rhTE and constructs containing domains 10–18 may have the potential to enhance EPC directed endothelial repair.

## Supporting Information

S1 Fig EPC proliferation on rhTE in the presence of antibodies to integrins α_2_β_1_, α_5_β_1_, and α_v_β_3_, applied at the time of cell seeding. These antibodies had no significant effect on EPC proliferation relative to the no antibody rhTE control. Error bars represent S.E.M. of triplicate measurements.
(TIFF)

S2 Fig ELISA specific for domain 6 of tropoelastin showing no significant differences in the relative amount of full-length rhTE and each of N25, N18 and N10. Error bars represent S.E.M. of triplicate measurements.
(TIF)
