# Supplementary figures and images for: Characterization of Endothelial Progenitor Cell Interactions with Human Tropoelastin
Source: PLoS One. 2015 Jun 26;10(6):e0131101. doi: 10.1371/journal.pone.0131101 (PMC4482626; doi:10.1371/journal.pone.0131101)

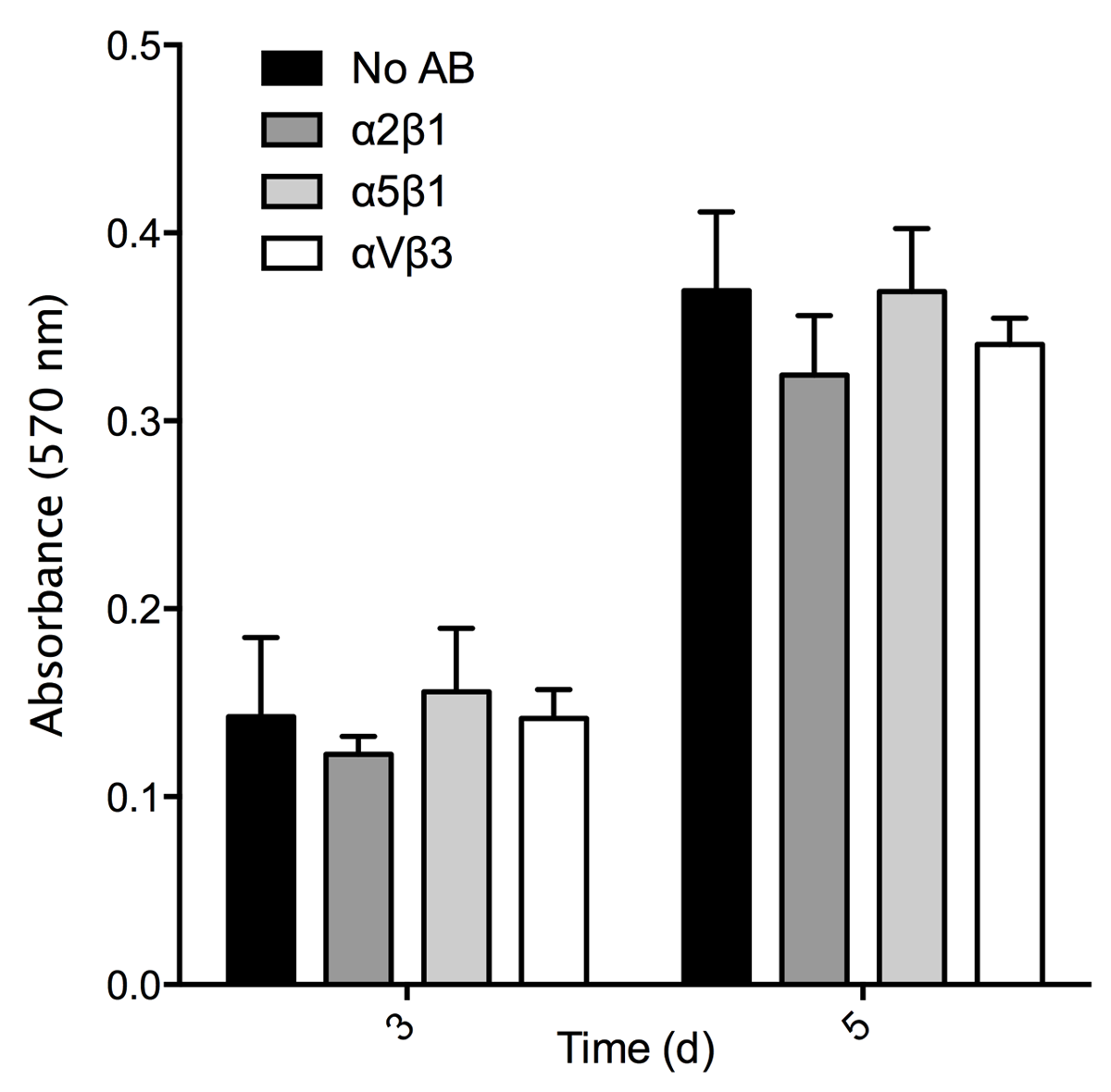

Supplement: S1 Fig — These antibodies had no significant effect on EPC proliferation relative to the no antibody rhTE control. Error bars represent S.E.M. of triplicate measurements. (TIFF) [file pone.0131101.s001.tiff]

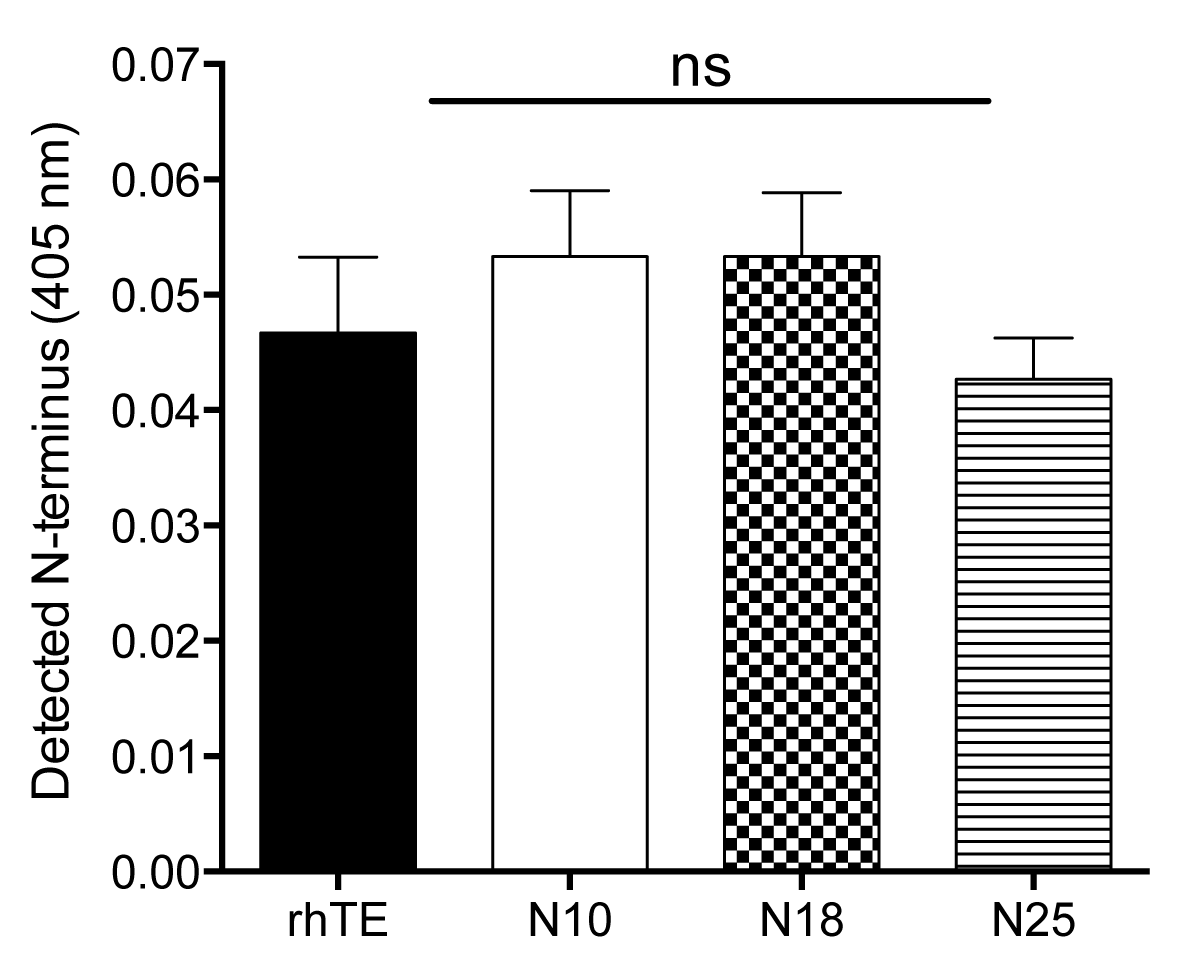

Supplement: S2 Fig — Error bars represent S.E.M. of triplicate measurements. (TIF) [file pone.0131101.s002.tif]
